# Gender-Dependent Effects of Enriched Environment and Social Isolation in Ischemic Retinal Lesion in Adult Rats

**DOI:** 10.3390/ijms140816111

**Published:** 2013-08-05

**Authors:** Peter Kiss, Krisztina Szabadfi, Gabor Horvath, Andrea Tamas, Jozsef Farkas, Robert Gabriel, Dora Reglodi

**Affiliations:** 1 PTE-MTA “Lendulet” PACAP Research Team, Department of Anatomy, University of Pecs, Pécs 7624, Hungary; E-Mails: peter.kiss@aok.pte.hu (P.K.); gabor.horvath@aok.pte.hu (G.H.); andreatamassz@gmail.com (A.T.); jozsef.farkas@aok.pte.hu (J.F.); 2 Department of Experimental Zoology and Neurobiology, University of Pecs, Pécs 7624, Hungary; E-Mails: kriszta.szabadfi@gmail.com (K.S.); gabriel@ttk.pte.hu (R.G.); 3 Janos Szentagothai Research Center, University of Pécs, Pécs 7624, Hungary

**Keywords:** retina, BCCAO, enriched environment, social isolation, gender

## Abstract

Exposure to an enriched environment has been shown to have many positive effects on brain structure and function. Numerous studies have proven that enriched environment can reduce the lesion induced by toxic and traumatic injuries. Impoverished environment, on the other hand, can have deleterious effects on the outcome of neuronal injuries. We have previously shown that enriched conditions have protective effects in retinal injury in newborn rats. It is well-known that the efficacy of neuroprotective strategies can depend on age and gender. The aim of the present study, therefore, was to examine the effects of environmental enrichment and social isolation in retinal ischemia. We used bilateral common carotid artery occlusion to induce retinal hypoperfusion in adult Wistar rats of both genders. Groups were housed in standard, enriched or impoverished conditions. Impoverished environment was induced by social isolation. Retinas were processed for histological analysis after two weeks of survival. In the present study, we show that (1) enriched environment has protective effects in adult ischemic retinal lesion, while (2) impoverished environment further increases the degree of ischemic injury, and (3) that these environmental effects are gender-dependent: females are less responsive to the positive effects of environmental enrichment and more vulnerable to retinal ischemia in social isolation. In summary, our present study shows that the effects of both positive and negative environmental stimuli are gender-dependent in ischemic retinal lesions.

## 1. Introduction

The most promising strategy to reduce the degree of neuronal loss in neuropathological conditions seems to be a combinatory approach [1–3]. Besides targeting apoptotic processes, neuroinflammation and oxidative stress with different drugs, environmental factors have been shown to have a major influence on the outcome of different neuronal lesions [4]. Environmental enrichment is a popular strategy to counteract the effects of neuronal injuries. Numerous studies have proven that enriched environment can reduce lesions induced by toxic [5–7], ischemic [8–10] and traumatic [11] injuries. The mechanism underlying this protective effect includes stimulating synaptic plasticity and neurogenesis, increase of dendritic spines and stimulating the expression of neurotrophic factors. As a consequence, improvement of cognitive and motor functions has been described [4,12–14].

Enriched environment has also been shown to influence the nervous system development, including that of the visual system [15,16]. Early postnatal and even prenatal enrichment have been shown to accelerate retinal development in rats [17,18]. Recently, we have shown, for the first time, that environmental enrichment has a protective effect in neonatal lesion of the retina [19,20]. In adult rats, we have studied the retinoprotective effects of several substances in lesions induced by several approaches, such as ischemia [21,22]. Ischemia is a leading cause behind numerous retinal pathologies leading to visual impairment [23]. The retinal degeneration caused by the compromise of retinal blood supply is the result of a complex cascade of harmful events. Therefore, protection against this lesion also calls for a complex approach, including anti-apoptotic, anti-inflammatory, anti-oxidant and other mechanisms [22]. Environmental enrichment exerts its protective effects at several levels and has already been shown to be protective in the retina [20,24,25]. Thus, it seems to be a good candidate for protection against retinal ischemic lesions. Since the plasticity of the adult nervous system is not comparable to the developing one, not surprisingly, many protective approaches effective in developing animals fail in adults [26]. The first aim of our present study was to examine whether enriched environment has protective effects in a model of retinal ischemia in adult rats.

In contrast to the protective effects of enriched environment, impoverished environment has been demonstrated to have deleterious effects in brain structure and function [27,28] and to worsen the outcome in several injuries [5]. Impoverished environment can be mimicked by housing rats in social isolation [4]. Isolation has been shown to cause, among others, an increase in the behavioral deficits and neuronal injury induced by kainic acid [5]. The retinal effects of social isolation are not well-known. We therefore sought to examine, as our second aim, the effects of social isolation in ischemic retinal lesions in adult rats.

It has long been known that the extent of neuronal injuries, as well as the efficacy of protective strategies can be gender-dependent [26,29,30]. In several models, females are usually less vulnerable to neurodegenerative insults, due to the increased endogenous neuroprotective effects of the female hormones. However, some of the neuroprotective treatments have been shown to be less effective in females than males [30]. Similarly, enriched and impoverished environmental conditions have been shown to have significant differences between male and female animals [31–35]. Therefore, the third aim of our study was to compare the degree of ischemic retinal lesion and the influence of environmental conditions in male and female rats.

## 2. Results and Discussion

**Figure 1:**
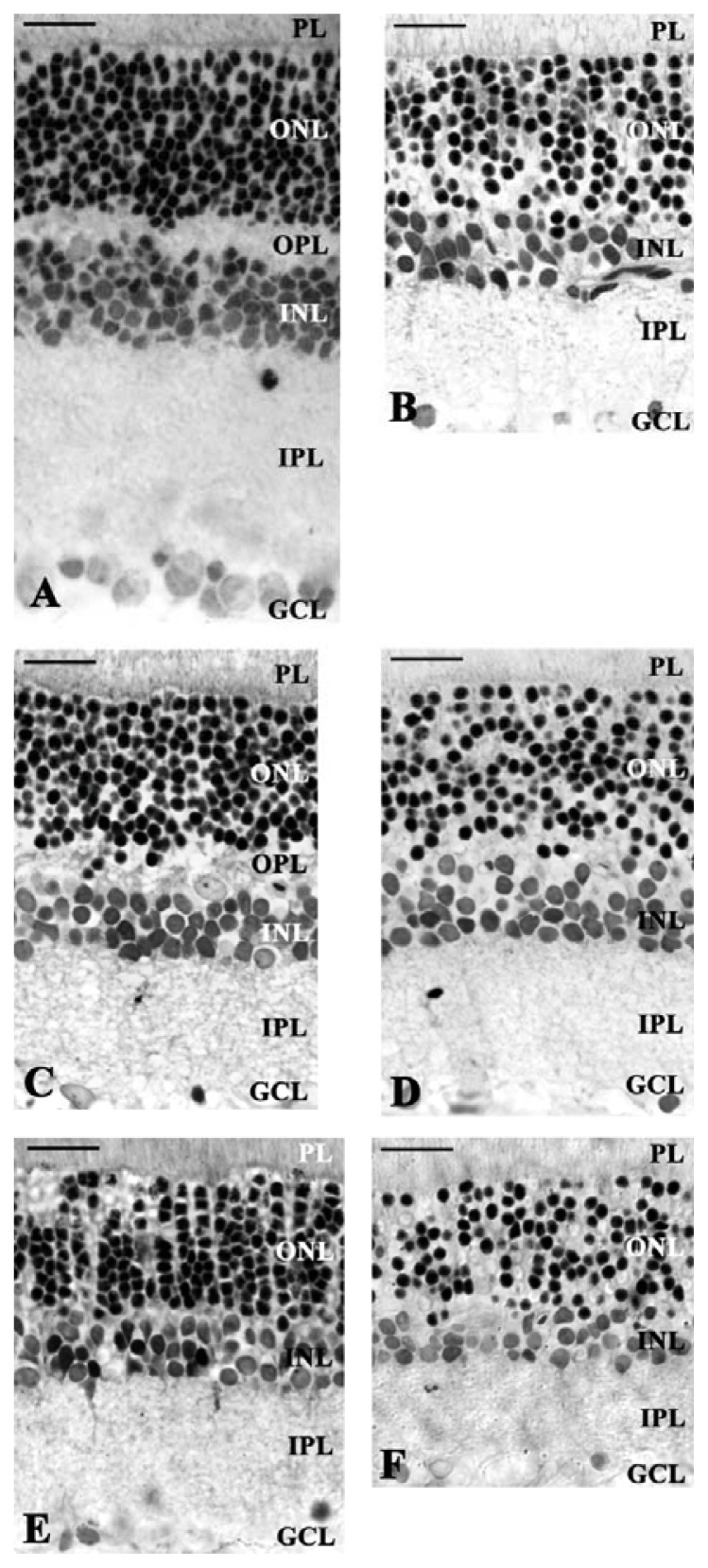
Representative microphotographs of toluidine blue-stained retinal sections derived from animals kept under different environmental conditions (standard housed, enriched environment and social isolation) from sham and bilateral common carotid artery occlusion (BCCAO)-operated male and female groups: (**A**) Sham-operated; (**B**) BCCAO-standard; (**C**) Male BCCAO with enriched environment; (**D**) Female BCCAO with enriched environment; (**E**) Male BCCAO with social isolation; (**F**) Female BCCAO with social isolation. Scale bar: 20 μm. Abbreviations: PL: photoreceptor layer; ONL: outer nuclear layer; OPL: outer plexiform layer; INL: inner nuclear layer; IPL: inner plexiform layer; GCL: ganglion cell layer.

Retinas of sham-operated animals showed normal appearance, similar to previous findings, comparable to intact animals (Figure 1A) [21]. All retinal layers were visible, such as the pigment epithelial layer, photoreceptor layer (PL), outer and inner plexiform layers (OPL and IPL), outer and inner nuclear layers (ONL and INL) and ganglion cell layer (GCL). There was no difference in retinal structure between male and female animals in this group (data not shown).

**Figure 2:**
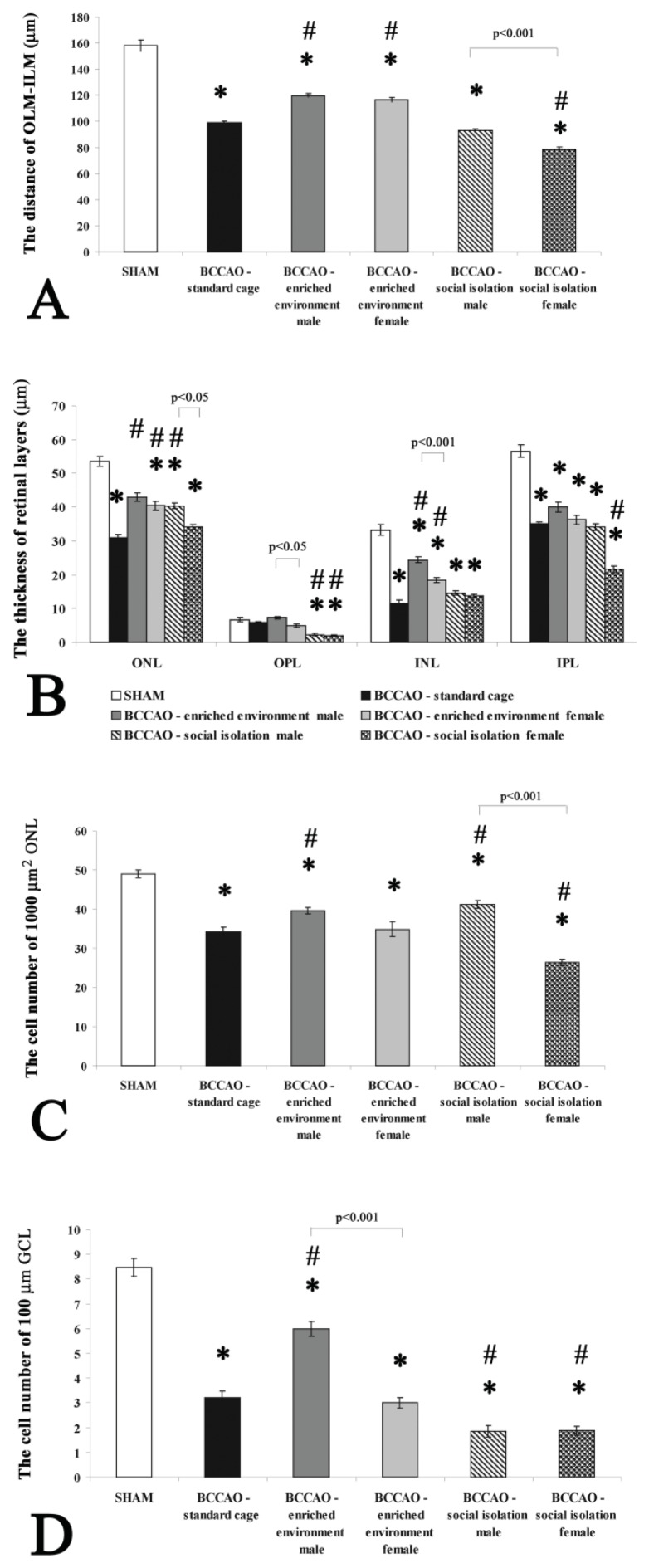
Morphometrical analysis of retinas from different groups of animals. Quantification of the thickness of the retina between OLM-ILM (**A**) thickness of layers, ONL, OPL, INL and IPL (**B**) cell number in 1,000 μm^2^ in ONL (**C**) and the cell number of 100 μm length of GCL (**D**). * *p* < 0.001 compared to sham-operated retinas; ^#^*p* < 0.001 compared to standard housed BCCAO retinas. Abbreviations: OLM: outer limiting membrane; ILM: inner limiting membrane; ONL: outer nuclear layer; OPL: outer plexiform layer; INL: inner nuclear layer; IPL: inner plexiform layer; GCL: ganglion cell layer.

Ligation of the carotid arteries for two weeks led to a severe degeneration in the retinal structure (Figure 1B), similar to our earlier observations [21,22]. Individual retinal layers were significantly reduced in thickness; the most marked reduction was observed in the INL and IPL (Figure 2B). As a consequence, the distance between outer and inner limiting membranes (OLM and ILM), corresponding to the entire retinal thickness, was significantly decreased (Figure 2A). Several structural abnormalities were also observed. In the nuclear layers (ONL and INL), numerous empty spaces were found between the intact neuronal perikarya. Numerous cells in the GCL also displayed severe degeneration (Figure 1B). This was well reflected in the reduced number of cells in the ONL and GCL (Figure 2C, D). No differences could be observed between the standard housed male and female bilateral common carotid artery occlusion (BCCAO)-operated groups (data not shown).

Retinas from rats kept under enriched conditions for two weeks following BCCAO showed a markedly better preserved retinal structure than retinas from the standard ischemic group (Figure 1B–D). This ameliorative effect of enriched environment could be observed in both males and females. Inner and outer nuclear retinal layers were less degenerated in both genders than in the standard ischemic group, resulting in a significantly greater OLM-ILM distance (Figure 2A,B). Enriched environment did not have a significant effect on the thickness of the plexiform layers (Figure 2B). In contrast to the standard ischemic groups, the effect of enriched environment resulted in being gender-dependent. The structure of the nuclear layers was not so well preserved in female animals. There was a tendency toward a smaller thickness in female retinas, reaching statistical significance in the OPL and INL. The empty areas could still be observed in the INL of female retinas, while they almost entirely disappeared in males (Figure 1C,D). The number of cells in the GCL of 100 μm retinal length was higher in the enriched environment BCCAO male group compared to the standard cage BCCAO retinas (Figure 2D). The biggest difference between male and female animals was observed in this parameter: no protection by enriched environment could be observed in females (Figure 2D). Similar differences were observed in the ONL: the number of cells in this layer was less in the ischemic female group than in the male animals (Figure 2C).

Slight differences in the retinal structure could be observed between the socially isolated male and standard housed groups after BCCAO (Figures 1B,E and 2). Retinal tissue from female rats with BCCAO housed in social isolation showed more severe degeneration compared to the male rats kept under the same conditions (Figure 1E,F). The morphometric analysis showed significant differences between the two groups in the distance of OLM-ILM and ONL (Figure 2A,B). The OPL thickness was significantly decreased in both of social isolated male and female groups compared to the standard housed BCCAO group (Figure 2B). The ONL and INL seemed intermingled with empty cell body-shaped holes in both layers (Figure 1E,F). The most severe degeneration was observed in the ONL, INL and IPL (Figures 1F and 2B). Quantitative analysis demonstrated significant differences in the GCL and ONL between the standard ischemic and social isolated groups (Figure 2C,D). Significant differences could be found in the cell number of ONL between the male and female social isolated groups. However, no statistical differences was observed in the cell number of GCL between males and females (Figure 2C,D).

In the present study, we showed that environmental conditions can influence the outcome of retina ischemic lesion in adult rats and that this effect is gender-dependent.

Our results prove that in spite of the reduced plasticity and regenerative potential of the adult nervous system, environmental stimuli can significantly modify the extent of ischemic retinal damage in adult rats. A lot of studies have shown that protective strategies effective in young animals fail to lead to morphological or functional improvement in adults. For example, a neuroprotective peptide highly effective in young males against nigral neurodegeneration has markedly less protective effects in aged rats [30]. Similarly, the effects of enriched/impoverished environment can be age-dependent [4,28,36]. However, enriched environment can be beneficial even in adult animals, as it has been described in several injury models and conditions [37,38].

A very recent study has also found that the outcome of retinal ischemia—caused by a different method—is improved by enriched housing in adult rats [25]. Our present results are thus in accordance with these findings and confirm the efficacy of enriched environment in ischemic lesion by another approach. Morphological improvement is generally accompanied by better functional recovery after different lesions [39], while functional improvement can be achieved with no obvious anatomical changes. This has also been described in enriched environmental paradigms. Martinez *et al.* [40] found that environmental enrichment influenced forelimb ability, but not tissue loss after traumatic cortical injury. However, the opposite has also been reported in the hippocampus: enhanced functional recovery was accompanied with increased ischemic death [41]. Whether the morphological amelioration observed in the present study is reflected in functional improvement cannot be concluded based on the present findings. However, our earlier results suggest that a similar degree of regeneration is accompanied by functional recovery confirmed by electroretinography [42]. The mechanism of enriched environment in hypoxia/ischemia-induced lesions has been investigated in other parts of the central nervous system. Zhu *et al.* [10] showed that the damaged plasticity induced by hippocampal hypoperfusion was enhanced by enriched environment in young adults. They described the involvement of increased pCREB, synaptophysin and MAP2 expression in this effect. Furthermore, specific changes in GABAergic and glutamatergic neurotransmission to increase synaptic strength and plasticity under enriched conditions have been described [37]. In addition, reduction of intracortical inhibitory mechanisms has also been described [43]. Furthermore, the involvement of MAP kinases, neurotrophic factors via the pathway, brain-derived neurotrophic factor (BDNF)/PI3K/GSK3beta, coupled with CREB activation has been reported in the brains of animals exposed to enriched environment [44]. Others have described that enriched environment exposure increased opioid signaling, acetylcholine release cycle and postsynaptic neurotransmitter receptors, but decreased Na^+^/Cl^−^-dependent neurotransmitter transporters, including dopamine transporter [45]. In the retina, the effect of enriched environment has been shown on trophic factors, like BDNF and insulin-like growth factor [15]. The molecular background in our model needs to be further clarified.

A major finding of the present study is that, in contrast to enriched environment, impoverished environment modeled by social isolation leads to more severe ischemic retinal degeneration. Social isolation is a widely-used model to study drug-induced behavioral changes, psychiatric disorders and stress conditions [46,47]. Very little is known about the retinal effects of impoverished environment. Priloff *et al.* [48] described that impoverished lighting conditions reduce the recovery capacity after optic nerve crush. Whether the social impoverished condition has any effect in retinal lesions has not yet been investigated. Our results are in accordance with those showing deleterious effects of social isolation in other injury paradigms, like kainic acid-induced seizures [5].

Gender differences have been shown in the pathophysiology and outcome in various neurological insults, such as ischemia, drug-induced neurotoxicity and neurotrauma [49–52]. Several lines of evidence suggest that gonadal steroids affect the onset and progression of neurodegeneration and the recovery from acute insults. Gender can also influence the efficacy of neuroprotective factors, although available data are very limited. Regarding ischemia, sex differences have been described in the brain [53]. Among others, a kappa opioid receptor agonist was protective in males, but not in female rats [54]. In a stroke model, male rats have been found to have a greater lesion than females, but benefited more from enriched environment afterwards [55,56]. Another study has reported that intervention with environmental enrichment after experimental brain trauma enhances cognitive recovery in male, but not in female, rats [57]. Arranz *et al.* [36] reported that male mice benefited more from enrichment exposure in a model of Alzheimer’s disease. Our present results are in accordance with these studies showing that environmental enrichment is not as protective in females as in males. The effects of social isolation have also been described to show gender-dependent differences [47]. Numerous studies have reported that females react more to social isolation. Arranz *et al.* [36] have described that females are more susceptible for enrichment removal. Others have also found that females are more sensitive to social isolation [35,58]. Our results are in agreement with these findings showing that females were more vulnerable to ischemic retinal lesion under impoverished conditions.

## 3. Experimental Section

### 3.1. Animals

Adult male and female Wistar rats (*n* = 49, 250–300 g, female estrus cycle synchronized) from a local colony were used for our experiments, as we have standardized the extent of ischemic retinal damage in these animals earlier. Animal housing, care and application of experimental procedures were in accordance with institutional guidelines under approved protocols (No. BA02/2000-15024/2011, University of Pecs). Food and water were available *ad libitum* and rats were kept under a 12 h light-dark cycle. Male and female rats were kept in separate cages.

### 3.2. Permanent Bilateral Common Carotid Artery Occlusion

Retinal ischemia was induced by permanent bilateral common carotid artery occlusion (BCCAO), a model of chronic cerebral hypoperfusion [10,59–61]. All steps were performed according to our earlier descriptions [21]. Under isoflurane anesthesia (Forane^®^, Aesica Queenborough Ltd., Kent, UK), both common carotid arteries were exposed through a midline cervical incision. Arteries were gently separated from the surrounding connective tissue and vagus nerve, then, they were ligated with a 3.0 filament. Wounds were closed using surgical stitches. A group of animals underwent anesthesia and all steps of the surgical procedure, except ligation of the carotid arteries (sham-operated controls, *n* = 7).

### 3.3. Environmental Enrichment and Social Isolation Paradigm

Immediately after BCCAO, animals were placed in different environments for 2 weeks. One group of rats was kept in standard cages with 43 × 30 × 20 cm (*n* = 7/male and *n* = 7/female, named as BCCAO-standard cage group). A second group of animals (*n* = 7 in both genders) was placed in enriched environment. This meant that the cages were larger (88 × 50 × 44) with a complex environmental enrichment. Rats were continuously exposed to intensive multisensory stimulation. The cage contained different toys, objects, running tunnels and rotating rods with various shapes, materials and colors. Half of the objects were changed daily, while the other half was left unchanged, according to our earlier descriptions [20]. A third group of rats (*n* = 7/male and *n* = 7/female) was kept in social isolation placed in a standard cage. Rats were placed individually in the cages, separated by 1 m from each other. All experiments were carried out at the same time, and animals were under the same illumination and other outside environmental conditions.

### 3.4. Histology

Two weeks after the BCCAO, rats were sacrificed under isoflurane anesthesia. The eyes were immediately dissected in ice-cold phosphate buffered saline and fixed in 4% paraformaldehyde dissolved in 0.1 M phosphate buffer (Sigma, Budapest, Hungary). Tissues were embedded in Durcupan ACM resin (Sigma, Budapest, Hungary), cut at 2 μm and stained with toluidine blue (Sigma, Budapest, Hungary). Sections were mounted in DPX Mountant for histology (Sigma, Budapest, Hungary) and examined in a Nikon Eclipse 80i microscope. Photographs were taken with a digital CCD type camera using the Spot program, from central retinal areas of nearly the same eccentricities (1–2 mm from the optic disc). Files were then further processed with the Adobe Photoshop 7.0 program. Samples for measurements were derived from at least six tissue blocks per animal (*n* = 4–5 measurements from one tissue block). The following parameters were measured: (i) cross-section of the retina from the outer limiting membrane to the inner limiting membrane (OLM-ILM); (ii) the width of the outer and inner nuclear and outer and inner plexiform layers (ONL, INL, OPL, IPL, respectively); (iii) the number of cells/100 μm section length in the ganglion cell layer (GCL) and the number of cells/1,000 μm^2^ in the outer nuclear layer (ONL). Results are presented as mean ± SEM. Statistical comparisons were made using the ANOVA test followed by Tukey-B’s *post hoc* analysis (* *p* < 0.001; ^#^*p* < 0.001). Due to the lack of statistical or morphological difference between genders in the sham and BCCAO-standard groups, results are not shown separately for male and female animals in these groups.

## 4. Conclusions

In summary, the present study showed that (1) enriched environment has protective effects in adult ischemic retinal lesion, while (2) impoverished environment further increases the degree of ischemic injury; and that (3) these environmental effects are gender-dependent: females are less responsive to the positive effects of environmental enrichment and more vulnerable to retinal ischemia in social isolation.
